# Changes in Care and Outcome for Children and Adolescents Living With HIV/AIDS During the COVID19 Pandemic in Germany—A Longitudinal Study

**DOI:** 10.1002/hsr2.71763

**Published:** 2026-01-19

**Authors:** Tobias Hante, Emilia Salzmann‐Manrique, Marla Braun, Stefan Schöning, Stephan Schultze‐Straßer, Björn Steffen, Eva Herrmann, Christoph Königs

**Affiliations:** ^1^ Goethe University Frankfurt, University Hospital Frankfurt, Department of Pediatrics and Adolescent Medicine Frankfurt Germany; ^2^ Goethe University Frankfurt, Department of Internal Medicine Frankfurt Germany; ^3^ Goethe University Frankfurt, Institute of Biostatistics and Mathematical Modelling Frankfurt Germany

**Keywords:** antiretroviral therapy, COVID19, pediatric HIV, retention in care

## Abstract

**Background and Aims:**

The COVID‐19 pandemic impacted daily life and healthcare. This study investigated how this affected the outcome in children and adolescents living with HIV/AIDS.

**Methods:**

This study investigated medical visits and outcomes (viral load, immunological parameters) in participants in the German Pediatric and Adolescent HIV cohort for 30 months, covering a period before and during the COVID‐19 pandemic.

**Results:**

Fifty‐two participants receiving antiretroviral therapy (27 females, 25 males; median age: 18 years, IQR: 15.0, 22.0) were analysed. Fifty percent of patients had fewer medical visits, 21% had an unchanged number of visits, and 29% had more medical visits when comparing the pandemic period with the prior period. (*p* = 0.016). This decline is primarily attributed to the first phase of the pandemic, where the incidence ratio of medical controls was 0.66 (95% CI 0.42–0.97) compared to the previous period (*p* = 0.05).

In male patients, there was a significant reduction in medical visits which was not seen in females. Among male patients, 60% experienced a decrease in control visits, while 20% remained unchanged (*p* = 0.015). Age of the patients was significantly negatively associated with number of visits (rho = −0.39; 95% CI [−0.63 to −0.15]; *p* = 0.004) before and during the pandemic (rho = −0.37; 95% CI [−0.58 to −0.12]; *p* = 0.007).

Half of the participants did not present with positive viral loads (≥ 20 cps/mL) in any of the two periods. Borderline cases of viral load measurements increased during the pandemic from 38% to 62% cases (*p* = 0.03), with the largest difference during the first wave.

**Conclusion:**

Overall, the study shows a negative impact during the COVID‐19 pandemic on the outcome in children and adolescents living with HIV in Germany and indicates the fragile nature of virological control in this population. Appropriate measures including virtual consultations should be implemented to improve outcome in future pandemic situations.

## 1 Introduction

After the first reports on a novel corona virus associated disease at the end of 2019, COVID‐19 cases increased dramatically around the world. In March 2020, the first restrictions were implemented in Germany to reduce the spread of SARS‐CoV‐2 including closure of schools, distancing measures and severe restrictions for participating in any activities. The national Robert Koch Institute defined four different phases of the pandemic from March 2020 to June 2021 based on the epidemiology, which also reflected the impact on daily life due to restrictions [1]. A study on mental well‐being after the first COVID‐19 wave (March 2020 to May 2020) including more than 1500 children, adolescents and their parents in Germany reported a significant burden in more than two thirds of the participants with a reduced quality of life and almost a doubling of children and adolescents with psychological problems [2].

With regards to medical care, access to hospitals and other medical institutions was also reduced. Staff was redeployed and premises were reorganized often leading to reduced capacities for non‐emergency health care provision—including the care of chronically affected children and adolescents. A survey in the UK found altered health care services in more than half of the Pediatric and Adolescent HIV centers, including 43% that extended the monitoring intervals for their patients. Respondents to this survey also reported difficulties in providing medical care, especially with regards to conversations with the young people and the involvement of a multidisciplinary team [3]. There are concerns that a reduction of care jeopardizes the success of care for adults and children living with HIV/AIDS (PLWHA, CLWHA) [4]. The relationship and cooperation of patients with the multidisciplinary team seems crucial for a successful care, adherence to therapy regimens and outcome [5].

In Germany, the UNAIDS targets for diagnosis, receiving therapy and having a successful therapy have been reached in 2021 including 96% of PLWHA on antiretroviral therapy (ART) had undetectable viral loads [6]. The number of undiagnosed HIV‐infections in children and teenagers in Germany is unknown but is expected to be only very few cases. In a cross‐sectional analysis performed in 2017, 348 children and teenagers (< 18 years) were identified in Germany, (GEPIC, Königs et al., conference abstracts). Out of these, 191 from four centers have been included so far into the *German Pediatric and Adolescent HIV Cohort* —GEPIC and followed longitudinally. All CLWHA received ART and 91% had undetectable viral loads (< 20 cps/mL) (Königs et al., unpublished).

So far, no data are available on the impact of the COVID‐19 pandemic on the retention in care and the success of treatment in Germany. Data mainly based on adults from 2021 suggest, that the percentage of PLWHA with successful therapy did not decrease in 2021 [6]. The portion of PLWHA presenting with advanced disease/AIDS at first diagnosis increased in Germany during the pandemic [7]. The seroprevalence of SARS‐CoV2 in adult PLWHA did not differ from the general adult population [8]. At the Frankfurt Pediatric HIV Treatment center, CLWHA are usually seen in person every 3 months. During the early days of the pandemic the capacity for outpatient visits was reduced by 50% (March and April 2020) due to redeployment of staff and reduced space following the implementation of measures related to the pandemic. Patients and parents were contacted by phone. No formal virtual outpatient clinic was established.

In order to understand the impact of the pandemic and changes due to the pandemic on the retention in care and outcome of CLWHA, data from the GEPIC cohort was analyzed 15 months prior to the pandemic (December 2018–February 2020) and during the first 15 months of the pandemic (March 2020–May 2021). Data on visits and virological and immunological outcome have been analyzed and compared.

## 2 Patients and Methods

### 2.1 Study Design and Population

The German Pediatric and Adolescent HIV cohort (GEPIC) is a national, multicenter, prospective cohort study. It includes children, adolescents and young adults with confirmed HIV infection followed at participating centers. GEPIC collects standardized data on demographic and clinical information, laboratory measurements, including anthropometric, virological and immunological parameters, cART and adverse events. GEPIC is based at the Goethe University, Department for Pediatrics and Adolescent Medicine in Frankfurt/Main. Data are documented by a central study coordinator visiting participating centers. The study has been registered in the German Clinical Trials Register (DRKS00007773).

For the current explorative analysis data were extracted and analyzed for a total of 30 months from December 1, 2018 to May 31, 2021 representing 15 months before and during the pandemic, respectively. Both periods were compared to each other. Additionally, the phases of the pandemic were considered and categorized into four different phases as defined by the national Robert Koch Institute as follows: first wave (March 10, 2020 to May 17, 2020), summer plateau 2020 (May 18, 2020 to September 27, 2020), second wave (September 28, 2020 to February 28, 2021) and third wave (March 01, 2021 to June 13, 2021) [6]. Results were analyzed based on the different phases as there were different impacts on daily life and also on medical care. All patients were included with a full data set for the entire period. As data entry were performed by a traveling study nurse, only data from the Frankfurt center were available due to travel and visit restrictions at the participating hospitals.

Epidemiological and clinical data were collected, as well as the medical visits performed and the results of laboratory tests. Medical visits were defined as in person visits at the outpatient unit. Lab examinations included viral loads (Abbott Alinity m HIV‐1 Assay, lower limit of detection: 20 cps/mL), absolute values and percentages of lymphocyte subsets: CD4+, CD8+, ratio CD4+/CD8+, CD3+, CD19+, and NK cells. Lymphocyte subsets were determined by flow cytometry as previously described [9]. C‐reactive protein (CRP), immunoglobulins IgA, IgG, IgM, triglycerides, cholesterol, HDL, LDL among others. Viral suppression was defined as viral loads < 20 cps/mL. Results were termed borderline positive when viral RNA was detected below 20 cps/mL (limit of detection), which could not be quantified. In addition, during the pandemic it was documented whether the patient had undergone IgG‐ or IgM‐COVID tests. Retention in care between the two periods is compared for the entire group as well as by sex, age, and ethnicity/country of origin.

### 2.2 Ethical Considerations

The study protocol and documents relevant for the study were submitted to the respective ethics committees. Central approval was obtained by the ethics committee of the Goethe University, Frankfurt (382/16). Patients and their caregivers signed an informed consent before inclusion to the study.

### 2.3 Statistical Analysis

Distribution of number of medical controls in the period before and during pandemic for each patient was compared using paired Wilcoxon signed‐rank test for the entire cohort as well as in subgroups by sex. Spearman's rho correlation analysis was used to examine the correlation between age and number of visits in each period.

Mixed effects binary logistic regression was performed to estimate the association between clinical and lab parameters and the impact of pandemic on viral load (pos/neg). Mixed effects Poisson regression and associated 95% confidence intervals (95% CIs) were used to estimate the impact of COVID‐19 phases on the incidence rate ratios (IRRs) of medical appointments occurring during the time of the study (Dec 2018 to May 2021). All mixed‐effects models considered a patient‐level random intercept.

With the aim to perform a longitudinal analysis of immunological laboratory markers before and during COVID‐19 pandemic a cubic B‐spline mixed effect regression model with two internal knots placed at the 33rd and 67th percentiles of the measurement times was used. The immunological profile analyzed in this study comprised the dynamics of absolute and relative values of CD4+, CD8+, ratio, CD3+, CD19+ and CD56+ NK cells. As well as other markers such as CRP, IgA, IgG, IgM, triglycerides, cholesterol, HDL and LDL. Data are presented as median with interquartile ranges. All tests were two‐sided and *p* values < 0.05 were regarded as statistically significant. Data analysis was performed by using R 4.2 software (http://www.R-project.org/).

## 3 Results

### 3.1 Demographics at First Visit

A total of 52 children and adolescents (27 female, 25 male) were included in this study. The median age was 18 years (IQR 15.0, 22.0). Most participant were of African ethnicity (50%), 36% were Caucasian and 14% African/Caucasian. The ethnicity of 10 study participants was unknown. All participants were receiving antiretroviral therapy, all with 2 NRTIs, 16 with an NNRTI‐based regimen, 12 with an INSTI‐based regimen and 24 with an PI‐based regimen. The viral load (median) was < 20 cps/mL (IQR: 0.0, 20.0). Forty‐four participants had undetectable viral loads. The median of absolute and relative CD4^+^ T cell count was 757/µL (IQR: 570.5, 982.0) and 37.25% (IQR: 29.0, 42.2), respectively.

### 3.2 Retention in Care

For each patient at least four visits were expected for each of the 15 months period. During the pre‐pandemic and pandemic period, 37 (71%) and 31 (60%) patients had at least four visits, respectively. The median number of the visits per patient was 4 (IQR: 3.0, 5.0) in the pre‐pandemic period and 4 (IQR: 3.0, 4.25) during the pandemic. The overall numbers of visits of the 52 included patients before the COVID‐19 pandemic (December 2018 until February 2020) was 218 and 187 during pandemic (March 2020–May 2021), which corresponds to a reduction of 14% in the second period.

**Figure 1:**
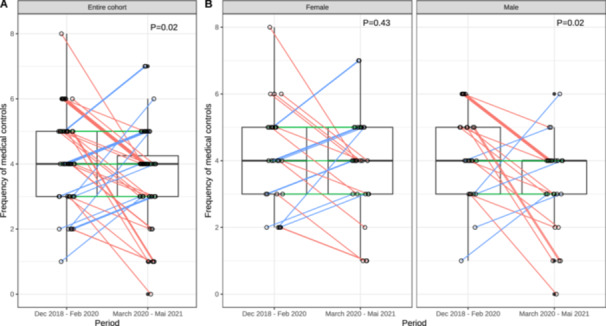
Notched box plots showing distribution of medical controls in the overall cohort and by sex. *p* values were calculated using two‐sided paired Wilcoxon signed‐rank test. (A) Entire cohort and (B) Female and male cohort.

In the entire cohort, 50% of patients had fewer medical visits, 21% had an unchanged number of visits, and 29% had more medical visits when comparing the pandemic period with the prior period. Medical visits decreased significantly between the two periods, with an effect size of 0.313 (*p *= 0.02; two‐sided paired Wilcoxon signed‐rank test). For male patients, the decrease was more pronounced, with an effect size of 0.490 (*p *= 0.02; two‐sided paired Wilcoxon signed‐rank test), while for female participants, no significant difference was found (*p *= 0.43; see Figure 1).

There were no significant changes based on age, ethnicity or country of origin when comparing both periods. For the total number of visits in both periods, there was a negative correlation between age and number of visits (rho = −0.39; 95% CI [−0.63 to −0.15]; *p* = 0.004) (rho = −0.37; 95% CI [−0.58 to −0.12]; *p* = 0.007). Pediatric patients significantly attended more doctor appointments than adolescent patients did.

**Figure 2:**
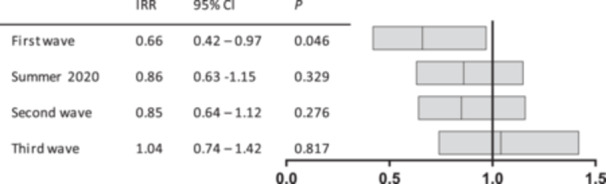
Incidence rate ratios are shown for visits during the different phases of the pandemic in relation to visits prior to the pandemic. *p* values were derived from mixed‐effects Poisson model, accounting for random intercepts at the patient level.

When looking at the different phases of the pandemic in Germany as defined by the national Robert Koch Institute [1], the significant decrease was seen during the first wave (March 10, 2020 to May 17, 2020). During the following three phases, the number of visits approached pre‐pandemic frequencies (Figure 2).

### 3.3 SARS‐CoV2 Serology

SARS‐CoV2 antibody testing was performed. SARS‐CoV2 specific IgG and IgM were measured. No testing was performed in the first wave, for IgG testing increased over the summer and third wave, whereas most IgM tests were carried out during the third wave. A total of 122 results for IgG were available. Seven patients were identified that had been in contact with the virus. Five of them tested positive during the second wave of the pandemic. All reported none or mild symptoms. No participant was hospitalized. For IgM, 72 test results were available. Again, seven participants tested positive for specific IgM.

### 3.4 Outcome of Clinical Care

#### 3.4.1 Viral Load Measurements

Viral load measurements were analyzed as an outcome parameter of clinical care. A total of 218 and 188 measurements were available for analysis before and during the pandemic, respectively. Before the pandemic 16% of tests (median: < 20 copies/mL, IQR: 0.0, 20.0) had viral loads above the cut‐off of 20 cps/mL and 19.6% (median: < 20 copies/mL, IQR 0.0, 20.0) during the pandemic. Half of the patients (26/52) did not have any viral loads above the cut‐off (HIV RNA ≥ 20 cps/mL) in any of the two periods. In 11 patients (21%) at least one viral load above the cut‐off was detected in both periods, whereas in 7 patients (13%), at least one viral load > 20 cps/mL was detected only in the period before COVID‐19 and in 8 patients (15%) during the pandemic.

**Figure 3:**
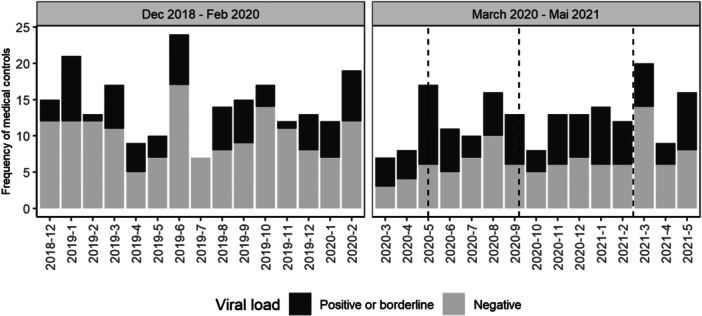
Bar plots showing the monthly frequency of medical controls. Black areas indicate positive or borderline viral load. Dashed lines indicate the different waves of COVID19 in Germany.

In addition, borderline positive viral loads were also analysed (detectable viral RNA at the detection limit, but not quantifiable). Before the pandemic 30% of all viral load results were positive or borderline positive, which significantly increased during the pandemic to 47% (*p *= 0.03). On a patient level, 19% of patients had at least one positive or borderline positive result in both periods, 15% only before the pandemic and 46% only during the pandemic. The number of viral load assessments per months divided into negative results and positive/borderline positive results are shown in Figure 3. There were no differences in positive or borderline positive viral load measurements based on the antiretroviral regimen.

### 3.5 Immunology

**Figure 4:**
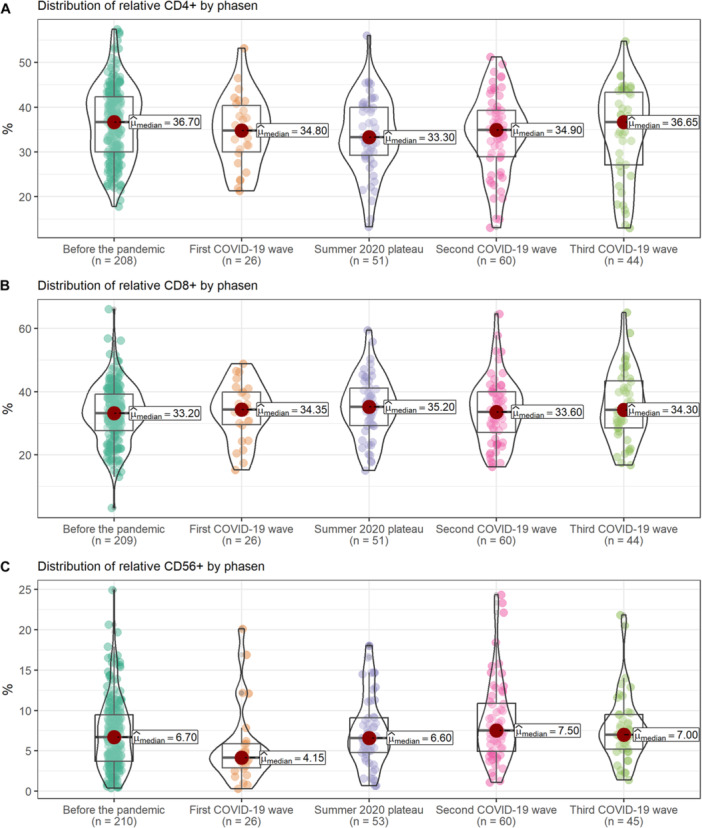
Relative distribution (%) of (A) CD4 cells, (B) CD8 cells, and (C) CD56 cells by wave.

During routine care lymphocyte subpopulations including CD4‐, CD8‐, CD19‐ and CD56‐positive cells were monitored. A total of 208 results were available for 52 patients in the 15 months before the pandemic and 182 of 52 patients during the first 15 months of the pandemic. The median CD4 cell count was 788/µL (IQR: 570.5, 982.0) and 782/µL (IQR: 564.8, 1001.0) before and during the pandemic, respectively with a relative CD4 portion of 36.7% (IQR: 29.03, 42.23) and 34.8% (IQR: 28.7, 40.6). As total numbers of lymphocytes and their subpopulations decrease during development in childhood and adolescents, relative cell counts were compared between the two study periods. A significant decrease of relative CD4‐positive cell numbers was observed during the pandemic: the percentage decreased by 1.92% (*p* = 0.02) and an increase in CD8‐positive cell numbers and portion. There was no difference for CD19‐positive cells. For CD56‐positive cells, a significant decrease was observed during the pandemic compared to pre‐pandemic times (*p *= 0.001) (Figure 4).

Analyzing the lymphocyte subpopulations according to the different phases of the pandemic, longitudinal differences were observed. For CD4‐positive cells, the biggest reduction was observed during the summer plateau compared to the pre‐pandemic period (−3%, *p *= 0.02). For CD56‐positive cells, the biggest reduction was observed in the first period. The results for the different subpopulations before the pandemic and during the different phases of the pandemic are shown in Figure 4A–C).

## 4 Discussion

Few data are available on the care of CLWHA and none from Germany regarding how the pandemic affected their care and outcomes. Data from the German Pediatric and Adolescent HIV cohort (GEPIC) were analyzed to describe and compare the situation of children and adolescents living with HIV/AIDS before and during the pandemic. Ninety‐seven percent of the enrolled participants were virologically suppressed (HIV RNA < 400 cps/mL) and 91% achieved viral loads < 50 cps/mL before the pandemic. European cohorts have reported viral load suppression (< 400 cps/mL) of 77% for children and teenagers or for adolescents transitioning into adult care of 60% [10, 11].

This study evaluated potential chances in care and outcome in GEPIC during the corona pandemic.

Not surprisingly a decrease of medical outpatient visits was observed during the pandemic. This decrease peaked during the first wave of COVID19 in Germany, when information on the virus and the pandemic were largely missing, strict restrictions were implemented, and routine visits were largely cancelled. Interestingly, the number of visits was reduced significantly in boys but not in girls. However, a study from Italy showed the opposite, where in this case adult female and not the male patients had reduced visits [12]. In the due course during the following phases of the pandemic the frequency of medical visits increased again. The health care system was not prepared for a pandemic and well established and successful routine care of chronically affected children and adolescents like in this cohort suffered. No alternative approaches including remote patient care were established. The impact of the pandemic on HIV care has been studied in various settings. A study conducted in Germany reported that the pandemic resulted in delayed diagnoses of HIV infection in adults [7]. No such data are available for children. A study conducted in Italy found a significant reduction in the number of patients accessing HIV care services during the pandemic, leading to poorer health outcomes for those living with HIV [13].

Regarding outcome of clinical care, a positive trend, but no significant differences were seen for viral loads above the local cut‐off of 20 cps/mL. Looking also at borderline positive results indicative for a low‐level viral replication, a significant increase of positive and borderline positive results were observed. Taking the cut‐off of 400 cps/mL, GEPIC participants achieved the UNAIDS goals of achieving negative viral loads in more than 95% of all patients before the pandemic, which was not achieved during the pandemic (93%).

The clinical impact of low‐level replication remains unclear. Nevertheless, a decrease in relative CD4‐cell numbers was observed. Again, it is unclear whether this decrease was due to a reduced adherence with low level replication and a reduced care or whether this was an epiphenomenon based on changes in antigen exposure following the implantation of infection control measures. In parallel to changes in CD4‐positive cells, changes in CD8‐ and CD56‐positive cells were observed. In terms of the impact on HIV viremia, an adult study conducted in Italy reported no significant difference in viral load among PLWHA during the pandemic [13].

Indisputably, the quality of care was hampered during the pandemic. As adherence remains relevant in care for CLWHA and PLWHA and the fact, that adherence is influenced by many factors, it may have been expected that reductions in care have a negative impact. Further, the pandemic has had a negative influence also on daily life of children and adolescents. The German COPSY study reported that children and adolescents experienced psychological distress during the first wave of the pandemic [2]. Further, it is established that the relationship of healthcare providers and patients does influence adherence in HIV care [5]. The British colleagues reported results from a survey emphasizing the potential impact of the pandemic on pediatric HIV care. The survey emphasized the potential impact and demands the analysis of the impact on COVID19 on pediatric HIV care [3]. The current GEPIC study gives further insights.

Surprisingly, until May 2021, only very few SARS‐CoV2 positive participants were identified in the cohort. The reasons for this observation are unclear. Potentially, the patients (and their parents) may have been more cautious to prevent infections and have implemented infection control measures, especially in the early months of the pandemic when little was known on the impact of COVID19 and PLWHA. Data from an adult cohort showed that was no difference in the prevalence of SARS‐CoV2 antibodies in plasma from PLWHA compared to a cohort living without HIV/AIDS [8].

Germany has a low prevalence in pediatric HIV infection. Therefore, the size of the analyzed cohort is small, which represents a limitation of this study.

The pandemic had and has further implication in HIV care worldwide. The number of children on antiretroviral therapy has declined in ten African countries [14]. Not only the quality of care and outcome was influenced, but also the prevention. In a pediatric context, the pandemic has affected the delivery of prevention of mother‐to‐child transmission (PMTCT) services, particularly among migrants. A study conducted in South Africa reported that healthcare providers faced challenges in providing PMTCT services to migrant women during the pandemic, leading to a decrease in the number of women accessing these services [15].

Outcome based on viral loads and CD4‐positive cell counts has been analyzed in the GEPIC cohort. The outcome during the pandemic demonstrated that therapeutic success was rather fragile. Measures need to be implemented to face challenges such as pandemics. Examples from other countries [12] may help to be better prepared for the future. This should focus on improved conversation [3] with CLWHA and their families, including virtual clinics with multidisciplinary teams, and should also incorporate contact via messaging services commonly used by the adolescent population.

## 5 Conclusions

Care for children and adolescents living with HIV has improved dramatically over the last two decades in Germany. The COVID19 pandemic has revealed the fragile status of this achievement. The establishment of services with a focus on improved and age‐appropriate conversations will be crucial to maintain a sufficient retention in care and a sufficient outcome in the future.

## Author Contributions

**Tobias Hante:** conceptualization, data curation, formal analysis, investigation, writing – original draft, writing – review and editing. **Emilia Salzmann‐Manrique:** formal analysis, methodology, visualization, writing – review and editing. **Marla Braun:** conceptualization, investigation, methodology, writing – review and editing. **Stefan Schöning:** conceptualization, methodology, resources, writing – review and editing. **Stephan Schultze‐Straßer:** funding acquisition, project administration, writing – review and editing. **Björn Steffen:** conceptualization, supervision, writing – review and editing. **Eva Herrmann:** formal analysis, methodology, supervision, writing – review and editing. **Christoph Königs:** conceptualization, formal analysis, funding acquisition, investigation, methodology, project administration, supervision, writing – original draft, writing – review and editing.

## Conflicts of Interest

The authors declare no conflicts of interest.

## Transparency Statement

The corresponding author, Christoph Königs, affirms that this manuscript is an honest, accurate, and transparent account of the study being reported; that no important aspects of the study have been omitted; and that any discrepancies from the study as planned (and, if relevant, registered) have been explained.

## Data Availability

All data are shown in the manuscript. Data on an individual patient level cannot be provided according to ethics approval. Only aggregated data can be shared, only in accordance with ethics approval and informed consent.
